# Supplementary figures and images for: Integrating multi-omics and machine learning to decipher the molecular pathways of bisphenol a-associated lactylation-related genes driving bladder cancer
Source: PLoS One. 2026 May 5;21(5):e0347134. doi: 10.1371/journal.pone.0347134 (PMC13143089; doi:10.1371/journal.pone.0347134)

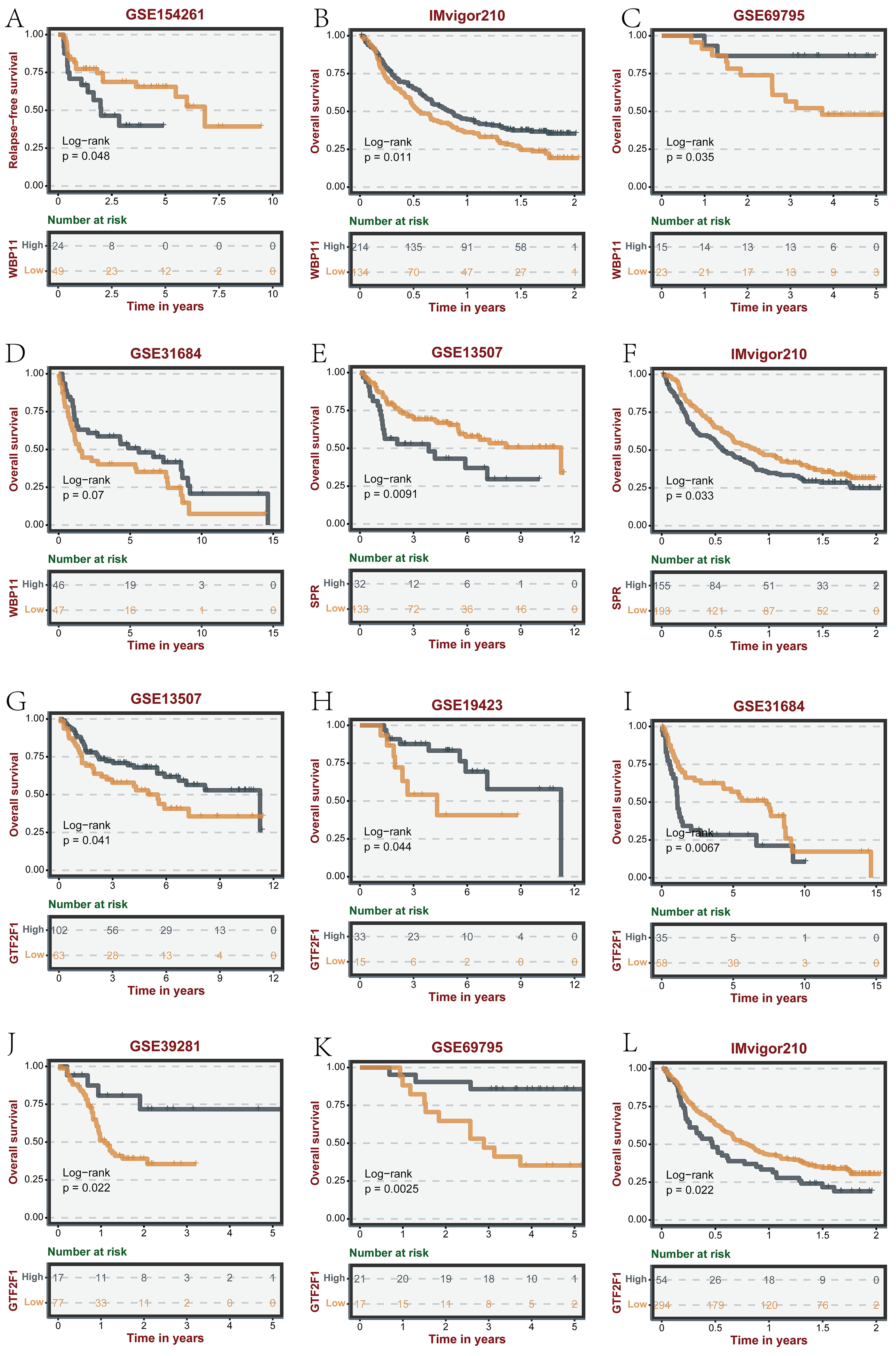

Supplement: S1 Fig — Kaplan–Meier survival curves for WBP11, SPR, and GTF2F1 in external validation cohorts. (TIF) [file pone.0347134.s001.tif]

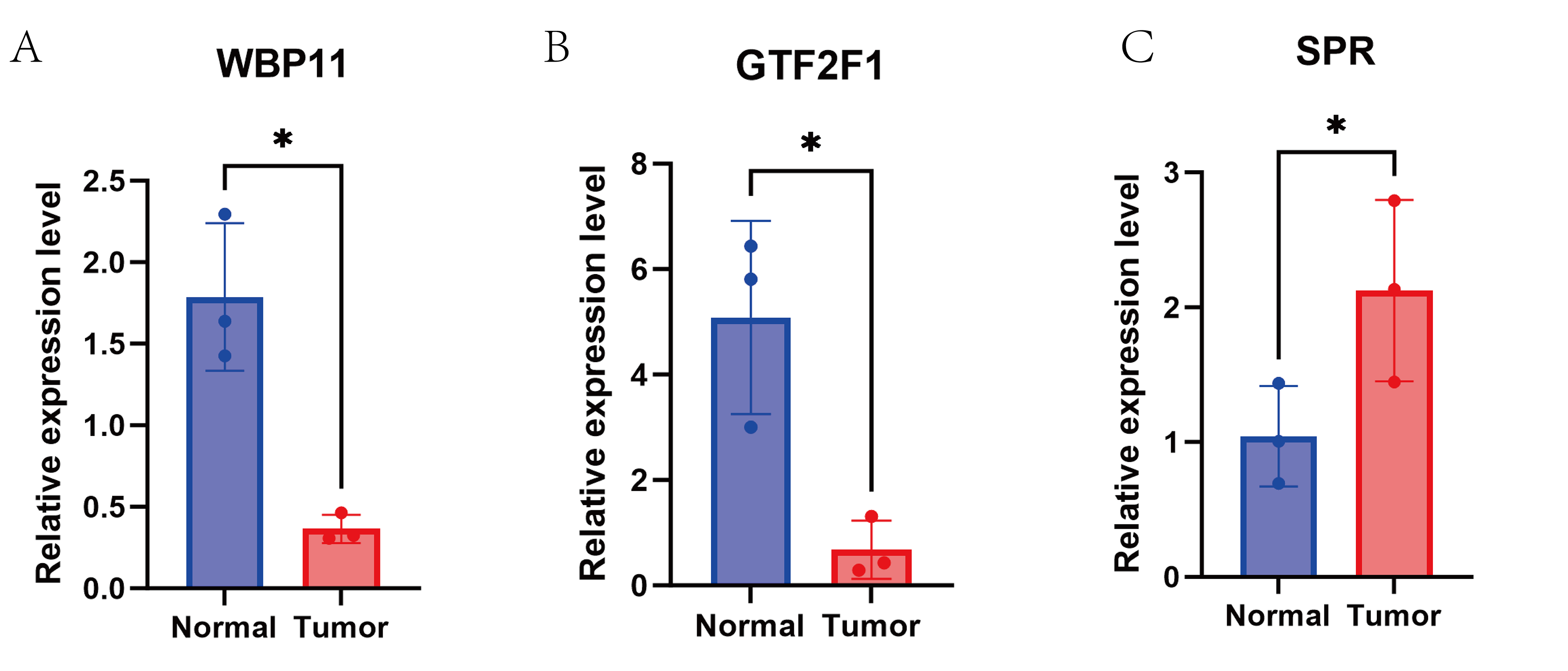

Supplement: S2 Fig — Relative mRNA expression levels of SPR, WBP11, and GTF2F1 were assessed by quantitative real-time PCR. (TIF) [file pone.0347134.s002.tif]

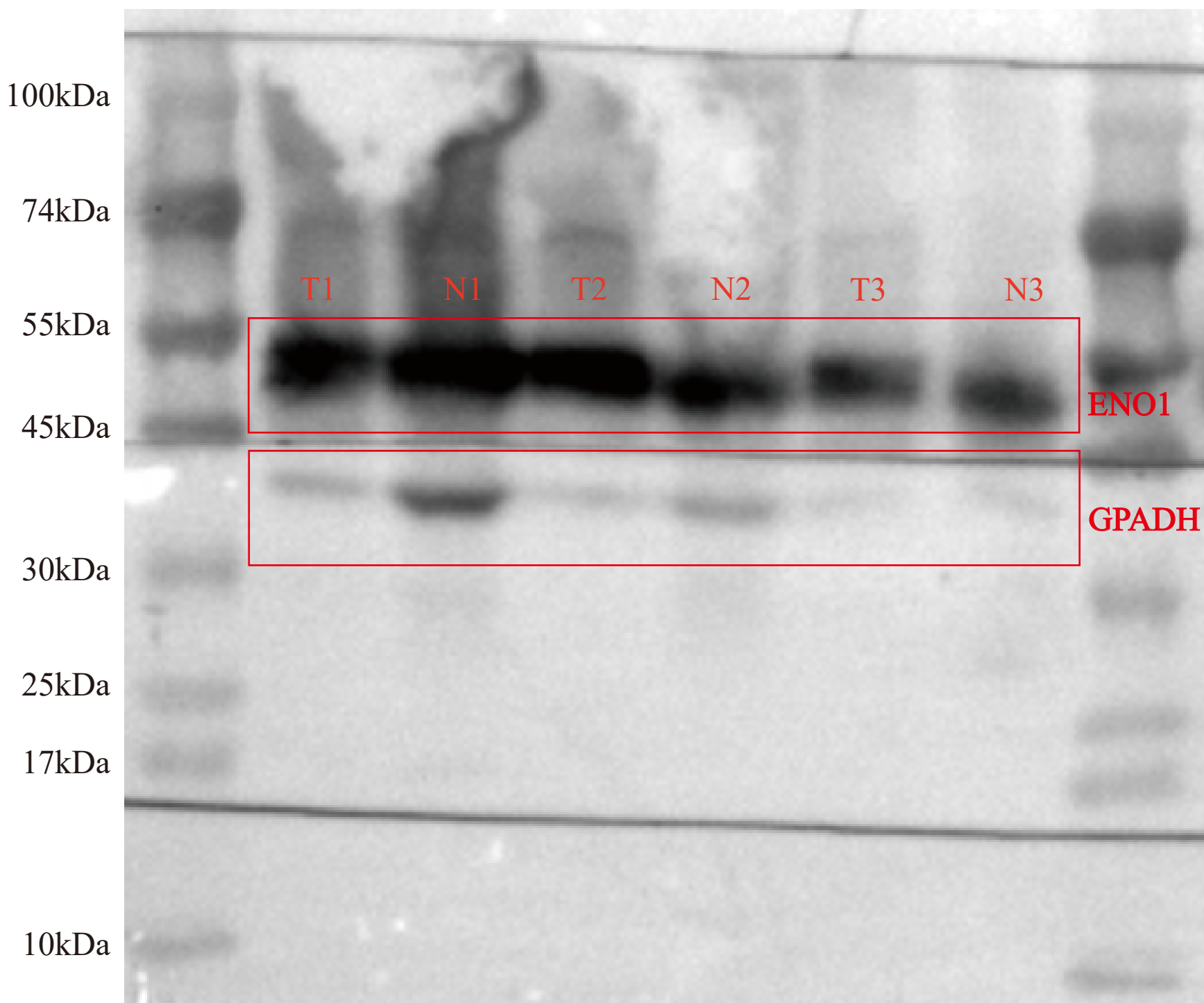

Supplement: S3 Fig — Full, uncropped western blot membranes used for ENO1 protein validation. (PDF) [file pone.0347134.s003.pdf]
