# Supplementary material for: Integrating multi-omics and machine learning to decipher the molecular pathways of bisphenol a-associated lactylation-related genes driving bladder cancer
Source: PLoS One. 2026 May 5;21(5):e0347134. doi: 10.1371/journal.pone.0347134 (PMC13143089; doi:10.1371/journal.pone.0347134)
Supplement: Attachment — Submitted filename: review_comments_25-67310.doc [file pone.0347134.s004.doc]

**Integrating Multi-Omics and Machine Learning to Decipher the Molecular Pathways of**

**Bisphenol A-Associated Lactylation-Related Genes Driving Bladder Cancer**

Wang et al, 2025

Major comments

1. The author used reliable sources from bioinformatics studies.
2. The aim of the study has not been clearly stated.
3. The clarity of the figures should be improved.
4. I appreciate the authors for incorporating the wet-lab results
5. The protein–protein interaction study reported 74 genes, clarify whether the four identified genes are included among them. If so, highlight it.
6. Explain clearly the evidence that supports the convergence and reliability of the 100 ns MD simulation in the study?
7. I encourage authors, the manuscript would benefit from extensive editing and proper formatting.
8. I suggest author to revise the manuscript to improve readability. The content is valuable and provides important insights for researchers.
9. I encourage author to provide a clarity in the conclusion to highlight the key outcomes and novelty of the study.

Decision: The manuscript has been accepted after the reviewers’ comments were addressed
