# Supplementary material for: Integrating multi-omics and machine learning to decipher the molecular pathways of bisphenol a-associated lactylation-related genes driving bladder cancer
Source: PLoS One. 2026 May 5;21(5):e0347134. doi: 10.1371/journal.pone.0347134 (PMC13143089; doi:10.1371/journal.pone.0347134)
Supplement: Attachment — Submitted filename: PONE-D-25-67310R1_review_comments.doc [file pone.0347134.s007.doc]

**Integrating Multi-Omics and Machine Learning to Decipher the Molecular Pathways of Bisphenol A-Associated Lactylation-Related Genes Driving Bladder Cancer.**

Jitao Wu et al.,

The author has addressed the requested questions.

Major comments:

1. The figures are still blurry and unclear. Please improve the clarity
2. I would encourage authors the alignment of the article needs to improve.
3. I suggest the authors explore ways to make the manuscript more reader-friendly, if feasible.
4. ENO1 has a well-known candidate in bladder cancer, the associations of WBP11, GTF2F1, and SPR with this disease have not been clearly established and should be justified by the author.
