# Supplementary material for: Integrating multi-omics and machine learning to decipher the molecular pathways of bisphenol a-associated lactylation-related genes driving bladder cancer
Source: PLoS One. 2026 May 5;21(5):e0347134. doi: 10.1371/journal.pone.0347134 (PMC13143089; doi:10.1371/journal.pone.0347134)
Supplement: Attachment — Submitted filename: Reviewer_comments_v01.docx [file pone.0347134.s005.docx]

PONE-D-25-67310: Integrating Multi-Omics and Machine Learning to Decipher the Molecular Pathways of Bisphenol A-Associated Lactylation-Related Genes Driving Bladder Cancer

Dear Editor,

Thank you for inviting me to review this manuscript. I appreciate the opportunity and am glad to contribute to the review process and support the journal’s work in maintaining strong academic standards. I will do my best to provide a careful and constructive assessment.

The authors present an interesting topic linking environmental exposure (BPA), epigenetics (lactylation), and bladder cancer. It is an ambitious framework that incorporates multi‑omics data and machine‑learning methods. They employ multiple layers of validation (bulk RNA, scRNA‑seq, immune infiltration, HPA, docking/MD), and the methods are largely standard, reproducible, and consistent with general practice. However, although the study is technically competent and addresses an important question, it overstates its conclusions, lacks direct evidence for lactylation and BPA–protein interactions, and requires substantial rewriting to better align its claims with the supporting data.

Major Concerns:

1) Overstated conclusions.

The manuscript implies causal relationships that are not fully supported by the data. All findings should be rewritten as predictive or hypothesis-generating, and a Limitations section must be added.

2) Lactylation not directly measured.

Protein lactylation is inferred from gene associations rather than modification-level evidence. The authors must clearly define “lactylation-related genes” and state this limitation explicitly.

3) Limited experimental validation.

Western blotting confirms protein expression only and does not validate lactylation or BPA–protein interactions. This distinction should be clearly stated throughout the manuscript.

4) BPA relevance requires clarification.

The link between BPA and bladder cancer is not firmly established. The authors should provide a balanced discussion and clarify that docking and simulation results do not imply in vivo interactions.

5) Docking and MD over-interpretation.

Molecular docking and MD simulations are described beyond their predictive scope. These results must be framed strictly as computational predictions.

6) Machine-learning validation concerns.

Batch correction, feature selection, and cross-validation procedures are insufficiently described. These methods must be clarified and performance claims tempered.

7) Immune infiltration over-interpretation.

Immune cell estimates are treated as precise measurements despite methodological variability. The authors should acknowledge limitations and focus on consistent trends.

8) A dedicated Limitations section must be added, explicitly acknowledging that experimental validation is limited to expression-level evidence.

9) Diagnostic and prognostic claims must be softened and clearly framed as exploratory rather than clinically actionable.

Minor Concerns:

1) Redundant Method descriptions.
2) Language imprecision and grammatical issues
3) Overly long sentences
4) Some unclear figure references
