# Supplementary material for: Integrating multi-omics and machine learning to decipher the molecular pathways of bisphenol a-associated lactylation-related genes driving bladder cancer
Source: PLoS One. 2026 May 5;21(5):e0347134. doi: 10.1371/journal.pone.0347134 (PMC13143089; doi:10.1371/journal.pone.0347134)
Supplement: Attachment — Submitted filename: Response to Reviewers.docx [file pone.0347134.s008.docx]

Dear Editor,

First of all, thank you very much for taking time out of your busy schedules to read and revise our manuscript. Thank you for your valuable comments. You have thoroughly advised us on the structure, content, research methodology and results of our paper. It has played a very important role in improving the quality of our paper, and on behalf of my co-authors, I would like to thank you for giving us the opportunity to revise and improve the quality of our article.

We have carefully read the reviewers' comments and your comments and have made changes, which are highlighted in yellow in the paper. We have tried our best to revise our manuscript based on the comments. and also fulfill the requirements of the journal. Attached is the revised version which we would like to submit for your consideration. We have written a point-by-point response letter for this purpose, the details of which are at the end of the letter.

In response to the journal's specific requirements, we have fully addressed each item: we have adjusted the manuscript to comply with PLOS ONE's style and file naming guidelines as per the provided templates; ensured all author-generated code is shared in line with best practices for reproducibility; updated the Funding Statement to include all sources of support and the required statement "There was no additional external funding received for this study"; clarified the funders' role in the study as specified; supplemented the Data Availability Statement with DOIs, accession numbers, and direct database links; re-uploaded high-clarity versions of Figures 1–16 following the journal's figure preparation guidelines; provided the original uncropped and unadjusted images for all blot/gel results as required.

Reviewer #1

**1.The authors present an integrative bioinformatics study combining public transcriptomic datasets, toxicogenomics annotations, immune deconvolution, single-cell resources, and molecular docking/MD to propose putative BPA-associated, lactylation-related drivers in bladder cancer. The topic is of potential interest; however, several major conceptual and methodological issues currently limit the strength of the central claims particularly the implied linkage between BPA exposure, lactylation biology, and bladder cancer pathogenesis. In its current form, the manuscript reads as a hypothesis-generating analysis rather than a biologically supported BPA–lactylation–cancer study.**

**Response:**

We thank the reviewer for this important clarification. We agree that the present study is primarily hypothesis-generating in nature. Accordingly, we have revised the manuscript throughout to explicitly frame our work as a toxicogenomics-informed and exploratory analysis aimed at prioritizing candidate genes and pathways, rather than establishing biologically validated causal mechanisms. Overly strong mechanistic or causal language has been systematically tempered, particularly in the Abstract, Discussion, and Conclusion.

**The revisions are in lines 29-32, 941-945, 1062-1089 of the revised manuscript**

**2.This study does not include patient-level BPA exposure measurements linked to the transcriptomic specimens. Consequently, the manuscript’s repeated “BPA-associated” framing is not evidence-based and appears to rest almost entirely on curated database annotations rather than cohort-specific data. In its current form, the work should be reframed as toxicogenomics-informed candidate prioritization. Alternatively, the authors must add direct exposure-linked analyses to substantiate any BPA–tumor association claims.**

**Response:**

We fully acknowledge this limitation. This study does not include patient-level BPA exposure measurements, and all BPA-related inferences are derived from curated toxicogenomics annotations in CTD. To address this concern, we revised the terminology throughout the manuscript (e.g., replacing “BPA-associated” with “BPA-linked candidates”) and explicitly clarified this limitation in the Methods, Discussion, and Limitations sections.

**The revisions are in lines 117-118, 893-904, 941-949 of the revised manuscript**

**3.** **Combining a large toxicogenomics-derived BPA target list with WGCNA modules and a literature-derived lactylation set is highly prone to producing seemingly "plausible" overlaps even under null conditions, particularly given gene-set size effects and network/module selection flexibility. The manuscript currently provides no rigorous statistical calibration. Without these analyses, the alleged specificity of the 74-gene intersection—and by extension the downstream “core gene” selection—remains unsubstantiated and may reflect methodological artifacts rather than biology.**

**Response:**

We thank the reviewer for this important methodological concern. We agree that intersecting large gene sets may produce seemingly plausible overlaps under null conditions, particularly due to gene-set size effects and module selection variability. To address this issue, we have added statistical calibration analyses in which random gene sets matched for size were repeatedly sampled to evaluate the expected distribution of intersection sizes under null conditions.

These analyses demonstrated that the observed 74-gene overlap exceeded random expectation and was not solely driven by methodological artifacts. In addition, we have revised the manuscript to clarify that the intersecting genes and the downstream core gene selection represent statistically prioritized candidates rather than definitive biological drivers, and we have appropriately tempered the related interpretations in the Discussion.

**The revisions are in lines 162-169, 486-488, 1062-1089 of the revised manuscript**

**4.** **While enrichment analyses may be consistent with a lactylation-related storyline, the manuscript offers no direct evidence of altered lactate metabolism/flux, no measurement of lysine lactylation marks, and no demonstration that BPA induces lactylation changes in bladder cancer-relevant systems. As written, the mechanistic interpretation is not warranted. The authors must either substantially temper the mechanistic claims or provide direct validation.**

**Response:**

We thank the reviewer for this important and constructive comment. We agree that the original version of the manuscript may have overinterpreted enrichment-based results in a mechanistic manner. As correctly noted, the present study does not include direct measurements of lactate metabolism or metabolic flux, does not assess lysine lactylation marks, and does not experimentally demonstrate BPA-induced lactylation changes in bladder cancer–relevant systems.

In response, we have substantially revised the manuscript to temper all mechanistic interpretations related to lactylation. Specifically, we clarified the definition of “lactylation-related genes” as genes functionally associated with lactylation-related pathways rather than proteins confirmed to be lactylated, revised the Results section to describe enrichment and association patterns without causal or mechanistic language, and explicitly acknowledged the absence of direct lactylation and metabolic validation in the Discussion and Limitations sections.

These revisions ensure that the lactylation-related findings are presented as transcriptomics- and enrichment-based functional associations within a toxicogenomics-informed framework, rather than as direct evidence of altered lactylation states or mechanisms.

**The revisions are in lines 566-570, 582-585 of the revised manuscript**

**5.** **Molecular docking and MD simulations can at best suggest structural plausibility of binding. They cannot establish biological activity, intracellular target engagement, downstream pathway modulation, or causal relevance to tumor phenotypes. Accordingly, any strong mechanistic claims derived primarily from docking/MD are not justified and should be removed or reframed as speculative, unless supported by orthogonal experimental evidence.**

**Response:**
We thank the reviewer for this important and constructive comment. We fully agree that molecular docking and molecular dynamics simulations can only provide evidence at the structural level and cannot establish biological activity, intracellular target engagement, downstream pathway modulation, or causal relevance to tumor phenotypes.

In response to this concern, we have carefully revised the manuscript to remove or substantially temper any mechanistic claims derived primarily from docking or MD analyses. The Results section now presents docking and MD findings strictly as evidence of structural plausibility and interaction stability, without implying functional or causal effects. In addition, we have revised the Discussion to explicitly clarify the evidentiary boundaries of these computational approaches and emphasized that any functional implications of BPA–protein interactions remain speculative and require independent experimental validation.

Furthermore, the limitations of molecular docking and molecular dynamics simulations are now explicitly acknowledged in the Limitations section. These revisions ensure that the interpretation of docking/MD results is fully aligned with their appropriate scope and level of evidence.

**The revisions are in lines 778-782, 822-831, 974-976, 1027-1026 of the revised manuscript**

**5.** **The manuscript refers to multiple “key genes,” yet experimental validation is provided only for ENO1. This selective validation weakens the claim that all identified candidates constitute robust key drivers/biomarkers.**

**Response:**
We thank the reviewer for this comment. We agree that experimental validation was performed only for ENO1. Accordingly, we have revised the Discussion section to explicitly distinguish ENO1 as the only gene supported by experimental validation, while the remaining genes are described as computationally prioritized candidates that warrant further experimental investigation. This revision ensures that the interpretation of “key genes” accurately reflects the different levels of supporting evidence.

**The revisions are in lines 893-900 of the revised manuscript**

**6.Figure 2: The resolution of the figures is too low to clearly read labels and interpret the content. Please provide higher-resolution versions of all figures in accordance with the journal’s image quality requirements.**

**Response:**
We thank the reviewer for this comment. Figure 2 has been regenerated and uploaded separately as a high-resolution version in accordance with the journal’s image quality requirements. The updated figure provides improved label clarity and overall readability.

**7.** **Line 636: The manuscript attributes the near-perfect training AUCs mainly to overfitting. Please elaborate on the likely causes of this overfitting and clarify what steps were taken to prevent information leakage and ensure unbiased performance estimates.**

**Response:**
We thank the reviewer for this important comment. The near-perfect training AUCs are primarily attributable to the high dimensionality of the transcriptomic features relative to the sample size, combined with the strong correlation structure among genes, which can lead to overly optimistic performance on the training data even when regularized models are used.

To mitigate overfitting and prevent information leakage, several precautions were taken. First, all feature selection and model training procedures were performed exclusively within the training set, and the validation datasets were kept completely independent and were not used at any stage of feature selection or model optimization. Second, cross-validation was applied within the training cohort to tune model parameters, and performance was assessed using held-out validation cohorts to obtain unbiased estimates. Third, consistent preprocessing steps were applied separately to training and validation datasets to avoid data leakage.

Accordingly, model performance was interpreted primarily based on validation and external cohort results rather than training AUCs. We have clarified these points in the revised manuscript to better explain the source of overfitting and the measures taken to ensure unbiased performance evaluation.

**The revisions are in lines 268-272, 279-281,665-661 of the revised manuscript**

**Additional comment**

**Line 47: Please define abbreviations at first mention by providing the full term followed by the abbreviation in parentheses.**

**Line 67: The wording “studies” appears inconsistent with the citations provided (only one reference is cited). Please revise for accuracy.**

**Line 79: Please spell out the full names of GEO and TCGA at first mention.**

**Line 482: Please check and standardize spacing in P-value reporting**

**Line 530: Please review and correct spacing/formatting issues.**

**Lines 732 and 737: Please check and correct spacing between words/sentences for readability.**

**Please review the manuscript to ensure consistent reference formatting throughout.**

**A comprehensive check is needed to ensure consistent abbreviation definitions and usage across the manuscript.**

**Response:**
We thank the reviewer for these helpful comments. The manuscript has been carefully revised accordingly. Abbreviations are now defined at their first mention, and the full names of GEO and TCGA are provided upon initial appearance. The wording at Line 67 has been corrected to reflect the appropriate number of cited references. Spacing and formatting issues, including P-value reporting and inter-word spacing at the indicated lines, have been corrected and standardized throughout the manuscript. In addition, we conducted a comprehensive review to ensure consistent abbreviation usage and reference formatting across the entire manuscript.

**The revisions are highlighted in yellow in the text**

**Reviewer #2**

**1.The author used reliable sources from bioinformatics studies.**

**Response:**
We thank the reviewer for this positive comment. We appreciate the recognition of the reliability of the bioinformatics data sources used in this study.

**2. The aim of the study has not been clearly stated.**

**Response:**
We thank the reviewer for this helpful comment. We agree that the study aim was not stated with sufficient clarity. In the revised manuscript, we have explicitly stated the aim of the study in the Introduction, clarifying that our objective was to systematically identify and prioritize potential BPA-associated, lactylation-related candidate genes in bladder cancer using integrative bioinformatics and multi-level validation approaches.

**The revisions are in lines 76-78 of the revised manuscript**

**3. The clarity of the figures should be improved.**

**Response:**
We thank the reviewer for this comment. All figures have been regenerated and uploaded as separate high-resolution files to improve clarity and readability. The revised figures now meet the journal’s image quality standards.

**4. I appreciate the authors for incorporating the wet-lab results**

**Response:**
We thank the reviewer for this positive comment. We appreciate the reviewer’s recognition of the inclusion of wet-lab experimental validation to support the bioinformatics findings.

**5. The protein–protein interaction study reported 74 genes, clarify whether the four identified genes are included among them. If so, highlight it.**

**Response:**
We thank the reviewer for this comment. Yes, all four prioritized candidate genes are included among the 74 genes in the protein–protein interaction network. These genes have been highlighted directly in the PPI figure to improve clarity and facilitate interpretation.

**6. Explain clearly the evidence that supports the convergence and reliability of the 100 ns MD simulation in the study?**

**Response:**
We thank the reviewer for this important comment. The convergence and reliability of the 100 ns molecular dynamics simulations were evaluated using standard stability metrics, primarily the root mean square deviation (RMSD) of the protein backbone and protein–ligand complexes. Following an initial equilibration period, the RMSD values reached a stable plateau and fluctuated within a narrow range during the latter portion of the simulations, indicating that the systems had achieved dynamic equilibrium.

In addition, complementary indicators such as RMSF and radius of gyration profiles showed consistent behavior over time, further supporting the structural stability of the simulated complexes. We have clarified these points in the revised manuscript to explicitly describe the evidence supporting the convergence and reliability of the MD simulations.

**The revisions are in lines 822-831 of the revised manuscript**

**7. I encourage authors, the manuscript would benefit from extensive editing and proper formatting.**

**Response:**
We thank the reviewer for this helpful suggestion. The manuscript has been carefully revised with extensive language editing and formatting improvements to enhance clarity, consistency, and overall presentation.

**8. I suggest author to revise the manuscript to improve readability. The content is valuable and provides important insights for researchers.**

**Response:**
We thank the reviewer for this encouraging comment and for recognizing the value of the manuscript. In response to this suggestion, we have revised the manuscript to improve readability, including language polishing, clearer presentation of the content, and improved overall organization.

**9. I encourage author to provide a clarity in the conclusion to highlight the key outcomes and novelty of the study.**

**Response:**
We thank the reviewer for this helpful suggestion. In the revised manuscript, we have added and refined a dedicated Conclusion section to more clearly summarize the key outcomes of the study and to highlight the novelty of the proposed toxicogenomics-informed analytical framework.

**The revisions are in lines 874-882 of the revised manuscript**

**Reviewer #3**

**1) Overstated conclusions.**

**The manuscript implies causal relationships that are not fully supported by the data. All findings should be rewritten as predictive or hypothesis-generating, and a Limitations section must be added.**

**Response:**
We thank the reviewer for this important comment. We agree that the findings of this study should be interpreted as predictive and hypothesis-generating rather than establishing causal relationships. In response, we have systematically revised the manuscript to temper any overstated conclusions and to consistently present the results as association-based and candidate-prioritizing.

In addition, we have added and refined a dedicated Limitations section to explicitly acknowledge the key constraints of the study, including the absence of patient-level BPA exposure data, the lack of direct measurements of lactate metabolism and lysine lactylation, the structural-level nature of molecular docking and molecular dynamics simulations, and the computational basis of immune infiltration analyses. These revisions ensure that the scope and conclusions of the study are fully aligned with the level of evidence provided.

**The revisions are in lines 1062-1086 of the revised manuscript**

**2) Lactylation not directly measured.**

**Protein lactylation is inferred from gene associations rather than modification-level evidence. The authors must clearly define “lactylation-related genes” and state this limitation explicitly.**

**Response:**
We thank the reviewer for this important comment. We agree that protein lactylation was not directly measured in this study and that lactylation-related findings are inferred from gene expression associations rather than modification-level evidence. In the revised manuscript, we have explicitly defined “lactylation-related genes” as genes functionally associated with lactylation-related pathways, rather than genes confirmed to carry lysine lactylation modifications. In addition, this limitation has been clearly stated in the Limitations section to ensure transparent interpretation of the results.

**The revisions are in lines 1062-1086 of the revised manuscript**

**3) Limited experimental validation.**

**Western blotting confirms protein expression only and does not validate lactylation or BPA–protein interactions. This distinction should be clearly stated throughout the manuscript.**

**Response:**
We thank the reviewer for this important comment. We agree that Western blotting in this study confirms protein expression levels only and does not validate lysine lactylation modifications or direct BPA–protein interactions. In the revised manuscript, we have explicitly clarified this distinction in the Results section describing the Western blot experiments. In addition, these limitations are clearly acknowledged in the Limitations section to ensure that the scope and level of experimental validation are accurately represented.

**The revisions are in lines 857-860, 1062-1086 of the revised manuscript**

**4) BPA relevance requires clarification.**

**The link between BPA and bladder cancer is not firmly established. The authors should provide a balanced discussion and clarify that docking and simulation results do not imply in vivo interactions.**

**Response:**
We thank the reviewer for this important comment. In the revised manuscript, we have provided a more balanced discussion clarifying that the relevance of BPA to bladder cancer remains incompletely established. Accordingly, we explicitly state that the findings should be interpreted as association-based and hypothesis-generating rather than as evidence of a direct causal relationship. In addition, we clarify that the intersecting genes identified in this study represent statistically prioritized candidates rather than definitive biological drivers.

**The revisions are in lines 893-900, 941-949, 1031-1034, 1062-1086 of the revised manuscript**

**5) Docking and MD over-interpretation.**

**Molecular docking and MD simulations are described beyond their predictive scope. These results must be framed strictly as computational predictions.**

**Response:**
We thank the reviewer for this important comment. We agree that molecular docking and molecular dynamics simulations are inherently predictive and should not be over-interpreted. In the revised manuscript, we have revised the Results and Discussion sections to explicitly frame all docking and MD findings strictly as computational predictions, without implying biological activity, functional interactions, or mechanistic causality.

**The revisions are in lines 778-782, 822-831, 1062-1086 of the revised manuscript**

**6) Machine-learning validation concerns.**

**Batch correction, feature selection, and cross-validation procedures are insufficiently described. These methods must be clarified and performance claims tempered.**

**Response:**
We thank the reviewer for this important comment. In the revised manuscript, we have clarified the machine-learning procedures in the Methods section, including data normalization within each cohort to mitigate potential batch effects, feature selection performed exclusively within the training dataset to prevent information leakage, and the use of a 5-fold cross-validation strategy combined with independent external validation.

In addition, we have tempered the interpretation of model performance throughout the manuscript, emphasizing the exploratory and predictive nature of the diagnostic models rather than making definitive performance claims. These revisions ensure transparent methodology and appropriately cautious interpretation of the machine-learning results.

**The revisions are in lines 268-272, 279-281 of the revised manuscript**

**7) Immune infiltration over-interpretation.**

**Immune cell estimates are treated as precise measurements despite methodological variability. The authors should acknowledge limitations and focus on consistent trends.**

**Response:**
We thank the reviewer for this important comment. We agree that immune cell infiltration estimates derived from computational deconvolution approaches are subject to methodological variability and should not be interpreted as precise quantitative measurements. In the revised manuscript, we have clarified that immune infiltration results represent computationally inferred relative trends rather than exact cell proportions.

In addition, we have revised the Results section to emphasize immune infiltration patterns that are consistent across multiple algorithms (MCPcounter, CIBERSORT, and ssGSEA), rather than focusing on absolute values. The methodological limitations of immune deconvolution analyses and the absence of experimental validation have also been explicitly acknowledged in the Limitations section. These revisions ensure a cautious and balanced interpretation of immune-related findings.

**The revisions are in lines 499-543, 1062-1086 of the revised manuscript**

**8) A dedicated Limitations section must be added, explicitly acknowledging that experimental validation is limited to expression-level evidence.**

**Response:**
We thank the reviewer for this important comment. In the revised manuscript, we have added a dedicated Limitations section explicitly acknowledging that experimental validation in this study is limited to expression-level evidence. We clearly state that post-translational modifications, direct BPA–protein interactions, and mechanistic effects were not experimentally assessed, ensuring transparent interpretation of the findings.

**The revisions are in lines 1062-1086 of the revised manuscript**

**9) Diagnostic and prognostic claims must be softened and clearly framed as exploratory rather than clinically actionable.**

**Response:**
We thank the reviewer for this important comment. We agree that diagnostic and prognostic findings in this study should be interpreted as exploratory rather than clinically actionable. In the revised manuscript, we have systematically revised the Discussion section to soften diagnostic and prognostic claims, avoiding definitive clinical or mechanistic language.

Specifically, we now frame ENO1 and other candidate genes as statistically prioritized and exploratory biomarkers rather than robust or clinically validated markers.In addition, molecular docking and simulation results are explicitly described as in silico structural predictions without implications for in vivo interactions.

These revisions ensure that all diagnostic and prognostic interpretations are fully aligned with the level of evidence provided and are not presented as clinically actionable conclusions.

**The revisions are in lines 893-900, 906-910,915-917, 1031-1034 of the revised manuscript**

**Minor Concerns:**

**1)	Redundant Method descriptions.**

**Response:**

We thank the reviewer for this comment. In the revised manuscript, we have carefully reviewed the Methods section and removed redundant descriptions to improve conciseness and avoid unnecessary repetition.

**2)	Language imprecision and grammatical issues**

**Response:**

We appreciate this suggestion. The manuscript has been thoroughly revised for language accuracy and grammatical correctness to improve clarity and readability.

**3)	Overly long sentences**

**Response:**

We thank the reviewer for pointing this out. Long and complex sentences have been shortened or restructured throughout the manuscript to enhance readability and ensure clearer presentation of the results and interpretations.

**4)	Some unclear figure references**

**Response:**

We thank the reviewer for this comment. All figure references have been carefully checked and revised to ensure clarity, consistency, and accurate correspondence between the text and the figures. We have increased the resolution of all images and uploaded them as separate files
