# Supplementary material for: Integrating multi-omics and machine learning to decipher the molecular pathways of bisphenol a-associated lactylation-related genes driving bladder cancer
Source: PLoS One. 2026 May 5;21(5):e0347134. doi: 10.1371/journal.pone.0347134 (PMC13143089; doi:10.1371/journal.pone.0347134)
Supplement: Attachment — Submitted filename: Response_to_Reviewers_auresp_2.docx [file pone.0347134.s010.docx]

Dear Dr. Pathak and Reviewers,

Thank you very much for your careful evaluation of our manuscript and for the constructive comments and suggestions. We sincerely appreciate the time and effort you and the reviewers devoted to assessing our work. These comments have been helpful in improving the clarity, rigor, and overall quality of the manuscript.

We have carefully considered all comments from the Academic Editor and reviewers and have revised the manuscript accordingly. In the revised version, all changes have been highlighted in yellow for ease of review, and a separate marked-up file with track changes has also been provided, in accordance with the journal’s requirements. In addition, we have prepared a detailed point-by-point response to each comment below.

During revision, we substantially improved the manuscript at both the scientific and presentation levels. Briefly, we refined the overall structure and readability of the text, clarified the interpretation of the study as a toxicogenomics-informed, hypothesis-generating prioritization analysis, improved figure quality by re-exporting and re-uploading all figures as high-resolution files, and added new supplementary analyses and experimental validation to strengthen the justification for the prioritized candidate genes. Specifically, we included additional survival analyses for WBP11, GTF2F1, and SPR across multiple independent cohorts (Supplementary Figure 1) and added tissue-level qPCR validation for these three genes (Supplementary Figure 2). We also revised the Discussion to more clearly distinguish the stronger evidence supporting ENO1 from the more exploratory status of WBP11, GTF2F1, and SPR.

We have also addressed the journal’s technical and reporting requirements in the revised submission, including revision of the manuscript files and figure files in accordance with PLOS ONE guidelines, updating the relevant reporting statements where needed, and providing the required supporting materials.

Reviewer

**1. The figures are still blurry and unclear. Please improve the clarity**

**Response:**

Thank you for your valuable suggestion. We have thoroughly improved the quality of the figures in the revised manuscript. All figures in the main text, together with the newly added supplementary figures included in this revision, were reassembled and exported as high-resolution images at 600 dpi to enhance sharpness, readability, and overall presentation quality. In addition, all revised figure files were uploaded separately in high-resolution format to ensure that image quality is preserved during review. We hope that these revisions have adequately addressed the reviewer’s concern.

**2.** **I would encourage authors the alignment of the article needs to improve.**

**Response:**

Thank you for this helpful comment. We have revised the manuscript to improve its overall alignment, coherence, and consistency. In the revised version, we streamlined the wording and structure of the **Abstract, Introduction, Results, and Discussion** so that the study is presented more consistently as a **toxicogenomics-informed, hypothesis-generating prioritization analysis** rather than a mechanistic or causal study. We also improved the correspondence between the figure order and the presentation of results in the main text, refined transitions between sections, and clarified the relative interpretation of the four prioritized genes. In particular, **ENO1** is now discussed as the gene with the strongest experimental support in this study, whereas **WBP11, GTF2F1, and SPR** are more clearly described as prioritized candidate targets supported mainly by integrative analyses and supplementary expression validation. We believe these revisions have substantially improved the alignment of the manuscript.

**3.** **I suggest the authors explore ways to make the manuscript more reader-friendly, if feasible.**

**Response:**

Thank you for this valuable suggestion. We have revised the manuscript to improve its readability at both the language and structural levels. In the revised version, we simplified several long or dense sentences, improved transitions between paragraphs and sections, and refined the presentation of key findings to make the overall narrative clearer and easier to follow. In addition, the manuscript was further polished with the assistance of a professional scientific editing service. For ease of review, all major readability-related revisions have been highlighted in yellow in the revised manuscript. We hope that these changes have improved the reader-friendliness of the article.

**The revisions are highlighted in yellow in the revised manuscript.**

**4.** **ENO1 has a well-known candidate in bladder cancer, the associations of WBP11, GTF2F1, and SPR with this disease have not been clearly established and should be justified by the author.**

**Response:**

Thank you for this important comment. We agree that, compared with ENO1, the associations of WBP11, GTF2F1, and SPR with bladder cancer are less well established in the current literature. In the revised manuscript, we have therefore clarified that these three genes should be interpreted as prioritized candidate genes identified through an integrative, toxicogenomics-informed screening framework, rather than as definitively established bladder cancer drivers.

Specifically, their inclusion was based on a multi-step prioritization strategy integrating WGCNA, BPA-related toxicogenomics targets, lactylation-related gene sets, and machine learning–based feature selection, through which WBP11, GTF2F1, and SPR were consistently retained together with ENO1. To further strengthen the justification for these three genes, we additionally performed survival analyses across multiple independent datasets. In particular, WBP11 was further evaluated in GSE154261, IMvigor210, and GSE69795; SPR was evaluated in GSE31684, GSE13507, and IMvigor210; and GTF2F1 was evaluated in GSE13507, GSE19423, GSE31684, GSE39281, GSE69795, and IMvigor210. These additional prognostic analyses have been added as Supplementary Figure S1.

In addition, we supplemented the study with tissue-level qPCR validation for WBP11, GTF2F1, and SPR, which provided preliminary experimental support at the transcriptional level for their inclusion as candidate genes. These qPCR results have been added as Supplementary Figure S2.

We hope that these additional analyses and textual revisions have adequately addressed the reviewer’s concern.

Supplementary Figure S1

Supplementary Figure S2

Thank you again for your time. We hope that the revised manuscript is now suitable for publication in PLOS ONE.

Sincerely,

Jitao Wu

on behalf of all authors
