# Supplementary material for: Integrating multi-omics and machine learning to decipher the molecular pathways of bisphenol a-associated lactylation-related genes driving bladder cancer
Source: PLoS One. 2026 May 5;21(5):e0347134. doi: 10.1371/journal.pone.0347134 (PMC13143089; doi:10.1371/journal.pone.0347134)
Supplement: Attachment — Submitted filename: PONE-D-25-67310R2.docx [file pone.0347134.s009.docx]

**Integrating Multi-Omics and Machine Learning to Decipher the Molecular Pathways of Bisphenol A-Associated Lactylation-Related Genes Driving Bladder Cancer**

**Jitao et al.,**

1. The author has made the manuscript more reader friendly.
2. I encourage authors to carefully review the manuscript formatting and alignment.
3. I encourage authors to improve the image quality, as some plots appear blurry. Example Figure1 the workflow and other example Figure 5 heatmap.

Decision: The author has addressed all comments; the manuscript will be accepted once all remaining review comments are appropriately addressed.
